# Comparative Sequence Analysis of the Non-Protein-Coding Mitochondrial DNA of Inbred Rat Strains

**DOI:** 10.1371/journal.pone.0008148

**Published:** 2009-12-07

**Authors:** Avinash Abhyankar, Hee-Bok Park, Giancarlo Tonolo, Holger Luthman

**Affiliations:** 1 Medical Genetics Unit, Department of Clinical Sciences-Malmö, Lund University, Malmö, Sweden; 2 Diabetology Unit, Azienda USL 2 Olbia, Olbia, Italy; Temasek Life Sciences Laboratory, Singapore

## Abstract

The proper function of mammalian mitochondria necessitates a coordinated expression of both nuclear and mitochondrial genes, most likely due to the co-evolution of nuclear and mitochondrial genomes. The non-protein coding regions of mitochondrial DNA (mtDNA) including the D-loop, tRNA and rRNA genes form a major component of this regulated expression unit. Here we present comparative analyses of the non-protein-coding regions from 27 *Rattus norvegicus* mtDNA sequences. There were two variable positions in *12S rRNA*, 20 in *16S rRNA*, eight within the tRNA genes and 13 in the D-loop. Only one of the three neutrality tests used demonstrated statistically significant evidence for selection in *16S rRNA* and *tRNA-Cys*. Based on our analyses of conserved sequences, we propose that some of the variable nucleotide positions identified in *16S rRNA* and *tRNA-Cys*, and the D-loop might be important for mitochondrial function and its regulation.

## Introduction

Mitochondria are the major energy producers in eukaryotic cells. Over millions of years of coexistence and coevolution, mitochondria have lost a considerable amount of their genome to the eukaryotic nuclear DNA [1], [2]. The mammalian mitochondrial DNA (mtDNA) encodes 37 genes, 13 of which form essential subunits of four mitochondrial respiratory chain complexes. The remaining genes for these complexes are encoded by the nuclear genome. Consequently, mitochondrial biogenesis, and hence function, needs an elaborate coordination of nuclear and mitochondrial gene expression [3], [4]. Apart from several ultra-short intergenic non-coding regions, mtDNA possesses a large non-coding D-loop that harbors regulatory regions for transcription and replication. The D-loop regulates mitochondrial replication and transcription in accordance with the energy demands, while the mitochondrial rRNAs and tRNAs ensure fulfillment of this task. Having its own genetic code different from the nuclear genetic code, mitochondria need their own protein biosynthesis system in the form of the mitochondrial ribosome (mitoribosome) built around 12S rRNA and 16S rRNA. The mitoribosome is responsible for the biosynthesis of the 13 proteins coded by the mtDNA and translates them with the help of 22 tRNAs also encoded by mtDNA. The non-protein-coding regions of the mtDNA are indispensable for cellular energy homeostasis, and genetic variation in these regions could have metabolic and fitness consequences. Since the protein-coding and the non-protein-coding regions of mtDNA serve different purposes – function and regulation of function – the variation pattern and the evolutionary pressures are expected to be different. Furthermore, the relative significance of coding sequence variation compared to the regulatory sequence variation, from an evolutionary perspective, remains poorly understood [5]. For this reason we investigated the protein-coding and the non-protein-coding regions separately. Here we present a molecular evolutionary analysis of the RNA genes and the D-loop of the rat mitochondrial genome. Information from 27 complete *Rattus norvegicus* mtDNA sequences was used.

## Results

### Ribosomal RNA Genes

**Figure 1:**
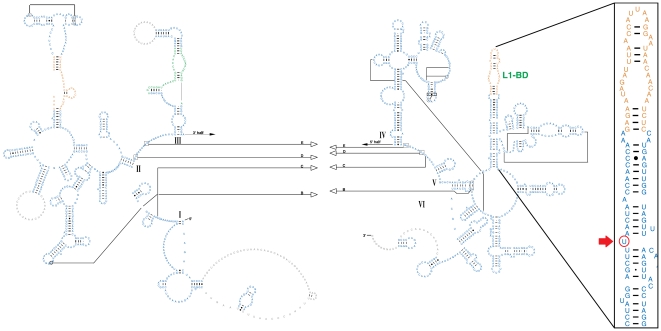
Location of variable position 2170 in the predicted secondary structure of the mammalian mitochondrial 16S rRNA. In the enlarged L1 binding domain, position 2170 is encircled and highlighted by a red arrow. L1-BD denotes the L1 binding domain. I, II, III, IV, V and VI represent the rRNA domains, while black arrows represent the predicted tertiary interactions. Blue color represents regions predicted from comparative sequence analysis, orange colour represents predictions by Mfold software, while green colour in domain III represents alternative secondary structure predicted using Alifold software. The figure has been modified from reference [6].

The mitoribosome is composed of a small subunit consisting of 12S rRNA and 29 proteins and a large subunit consisting of 16S rRNA and 58 proteins [6]. Comparison of the 27 rat mtDNA sequences (Table S1) revealed seven variable positions in *12S rRNA*, five of them unique to the wild rats (Table S2). Excluding the five variant positions unique to the wild rats, only positions 935 and 942 were considered for further analysis. None of these two variable positions alter the predicted 12S rRNA secondary structure or the free energy estimates. Mapping of these two sites on the consensus secondary structure for mammalian mitochondrial 12S rRNA showed that they are located in the 3′ minor domain [7]. However, we could not find any conservation at these two positions when compared to nine different mammalian species (data not shown). In *16S rRNA* there were 23 variable positions, 20 of those were found among the inbred strains, while three variable positions were unique to the wild rats (Table S2). Within *16S rRNA*, we noted a poly-C tract starting at position 1131 varying between five and eleven cytosines. Six of the variant positions were located in this poly-C tract. Taken together in haplotypes, the variant positions within *16S rRNA* affect the topology and free energy estimates of the predicted secondary structures. We also assessed the conservation pattern for these variants using multiple alignments of nine different mammalian mitochondrial sequences. Of the variable positions in *16S rRNA* only position 2170 was conserved among mammalian species; this C to T substitution is located in a 28-nucleotide long conserved sequence in close proximity to the L1-binding domain (Figure 1).

### Transfer RNA Genes

**Figure 2:**
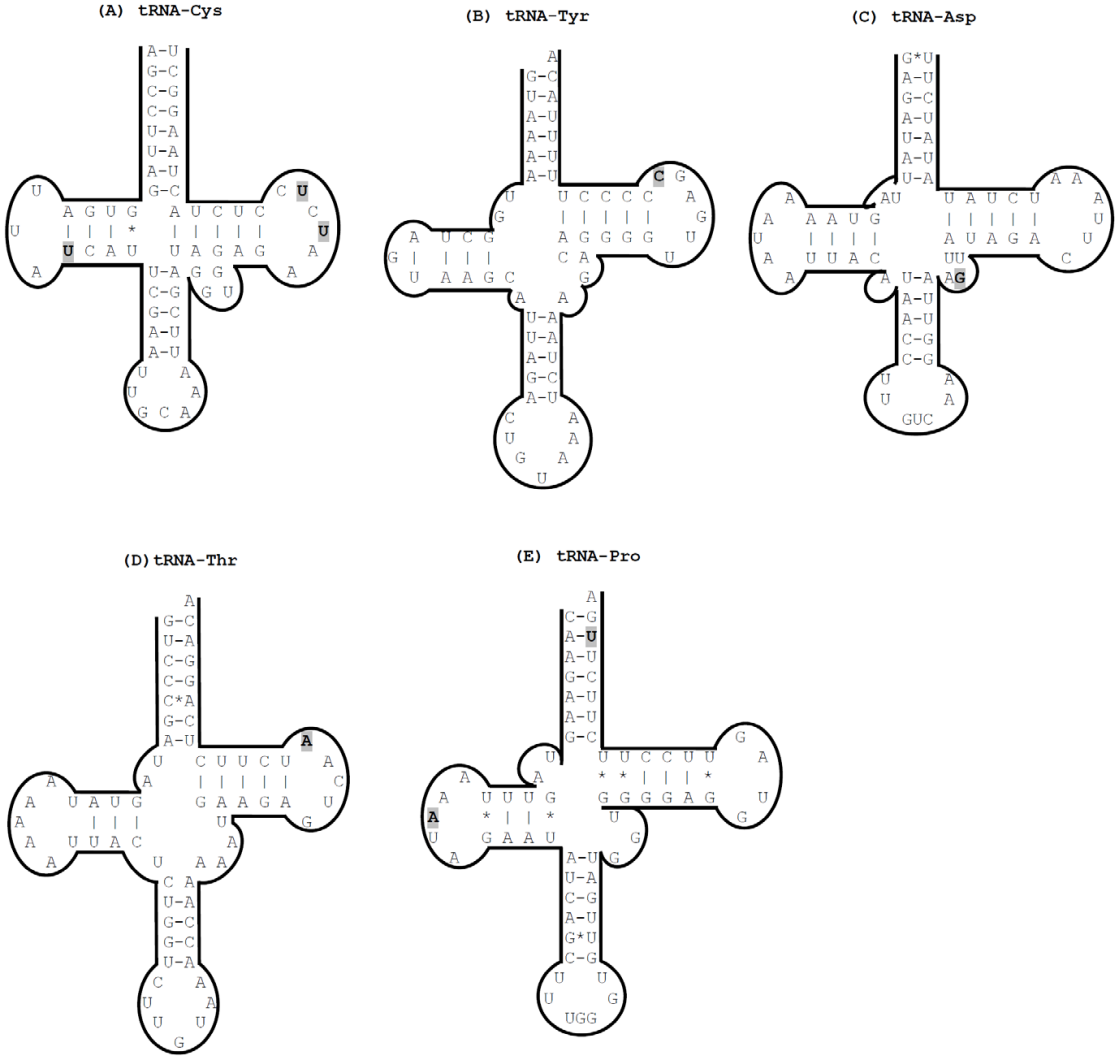
Inferred secondary structures for (A) *tRNA-Cys*, (B) *tRNA-Tyr*, (C) *tRNA-Asp*, (D) *tRNA-Thr* and (E) *tRNA-Pro*. Variable sites are highlighted in grey. The anticodon loop appears at the bottom of each model.

The comparative analysis of the 22 tRNAs in mtDNA revealed a high degree of conservation. Only five of the 22 tRNAs had variable sites occurring in more than one strain (Table S2). All singletons were attributed to the wild rat sequences, except one at position 15350 that was unique to the WKY/NCrl strain. Three variable sites were observed in *tRNA-Cys* and two in *tRNA-Pro*, while *tRNA-Tyr*, *tRNA-Asp* and *tRNA-Thr* had one variable site each. There was a clear grouping pattern of the Wistar-derived and non-Wistar derived strains of the three variable positions in *tRNA-Cys* (positions 5200, 5202 and 5237). All strains originating from the Wistar rat (Table S2) shared the same allele at all these three positions indicating inheritance of an ancestral haplotype. At position 5202 the ‘Wistar’ allele was also shared by three wild rats – Wild/Cop, Wild/Tku and Wild/Mcwi. A similar Wistar-specific grouping was seen for the remaining four variable tRNA genes (*tRNA-Tyr*, *tRNA-Asp*, *tRNA-Thr* and *tRNA-Pro*). To assess the structural implications of these variants, we modeled their secondary structures based on consensus secondary structures of the mammalian mitochondrial tRNAs [8]. Figure 2 shows our secondary structure models for all the five tRNA genes.

### The D-Loop

The only major non-coding region of the mitochondrial DNA is the D-loop. A total of 13 variable sites were found in the D-loop of inbred strains – eleven substitutions and two insertion/deletions. We mapped the known D-loop functional sites to the rat mitochondrial sequence [9], [10], [11], [12] (Table 1). Six substitutions were located in the termination associated sequences (TAS, ETAS), one substitution in the central block (CB), while the conserved sequence block 2 (MT-CSB2) had one insertion/deletion. Position 15460, located in ETAS1 deserves special attention since it is not only conserved between various *Rattus* species (*R.rattus, R.exulans, R.tiomanicus, R.hoffmanni, R.tanezumi*, and *R.sordidus*) but also in nine different mammalian species. The last nucleotide of the D-loop was also variable.

**Table 1:** Functional sites in the D-loop of rat mtDNA.

Locus	Description	Start (bp)	End (bp)	Variants
**ETAS1**	Termination-associated sequence	15446	15503	1 substitution
**TAS-D**	Termination associated sequence	15497	15511	1 substitution
**TAS-C**	Termination associated sequence	15520	15531	0
**TAS-B**	Termination associated sequence	15541	15554	1 substitution
**TAS-A**	Termination associated sequence	15571	15584	1 substitution
**ETAS2**	Termination-associated sequence	15511	15572	2 substitutions
**CB**	Central Block	15673	15979	1 substitution
**MT-OHR**	H-strand origin	16026	16026	0
**MT-CSB1**	Conserved sequence block 1	16027	16052	0
**MT-CSB2**	Conserved sequence block 2	16083	16099	1 insertion/deletion
**MT-CSB3**	Conserved sequence block 3	16116	16133	0
**MT-LSP**	L-strand transcript initiation site	16193	16193	0
**MT-TFL**	Tfam binding site	16212	16226	0
**MT-TFH**	Tfam binding site	16267	16286	0
**MT-HSP1**	H-strand transcript initiation site	16298	16298	0

Nucleotide numbering represents position in the BN/SsNHsdMCW mtDNA sequence (Acc. No. NC_001665).

### Tests for Selection

The Tajima's D test and the Fu & Li's D and F tests were performed on all the RNA genes and the D-loop to assess any deviation from neutrality. Since the results of these tests would also be influenced by population size changes, we estimated the F_S_ and R2 statistics but did not find support for such population changes (data not shown). We found evidence for selection in *16S rRNA* and *tRNA-Cys* based on Tajima's D test, whereas Fu and Li's D and F tests did not provide any evidence for selection in the RNA genes or in the D-loop (Table 2).

**Table 2:** Summary statistics for selection analyses in the non-protein-coding mtDNA.

Feature	No. variable sites	No. haplotypes	Diversity per site	Tajima's D	Fu & Li's D	Fu & Li's F
**12S rRNA**	3	2	0.001	2.037	0.858	1.35
**16S rRNA**	20	8	0.003	2.56**	0.765	1.48
**tRNA-Cys**	3	3	0.022	2.323*	0.62	1.00
**tRNA-Tyr**	1	2	0.007	1.566	0.642	1.01
**tRNA-Asp**	1	2	0	1.566	0.62	1.00
**tRNA-Thr**	1	2	0.007	1.505	0.642	0.99
**tRNA-Pro**	2	3	0.003	−1.164	−1.558	−1.66
**D-loop**	12	6	0.003	1.334	1.105	1.10

All estimates exclude singletons observed in the sequences from wild rats.

Tajima's D = Results of Tajima's D test.

Fu & Li's D = Results of Fu & Li's D test with outgroup.

Fu & Li's F = Results of Fu & Li's F test with outgroup.

* = *P*<0.05.

** = *P*<0.01.

## Discussion

Mitochondrial DNA encodes few but essential components of the respiratory chain complexes I, III, IV and V. The two ribosomal RNAs provide a scaffold for the mitochondrial ribosomal proteins (MRPs). The mammalian mitoribosome has significantly reduced RNA content as compared to its bacterial counterpart; this reduction is accounted for by an increase in the number of MRPs [13], [14]. This reduction exerts strict structural constraints on the ribosomal RNAs for efficient and accurate function. In bacteria and archea the ribosomal protein L1 has a dual function as a ribosomal protein binding 23S rRNA and as a translational repressor by binding mRNA [15], [16]. The L1 binding domain in the mammalian mitochondrial *16S rRNA* was found to be highly conserved [17]. According to our analysis, only one of the observed variable positions in the rat *16S rRNA* (position 2170) is highly conserved and might be of functional importance due to its close proximity to the L1 binding domain (Figure 1).

Out of the 22 tRNA genes only five had variant positions among the 27 investigated rat sequences. According to our prediction, *tRNA-Cys* variant A5202G could potentially have a destabilizing effect on its secondary structure and compromise the efficiency of cystein incorporation in a growing peptide chain. Stem-loop structures in the vicinity of the L-strand origin are also important for accurate and efficient replication of mtDNA [18], [19], [20]. Two of the three *tRNA-Cys* variants (positions 5200 and 5202) are located in these loop structures. Taken together, the observed variation in the rat mitochondrial *tRNA-Cys* might not only affect the role as a tRNA but also affect priming of L-strand replication.

Mitochondria have an unusually high capacity for initiation of DNA replication, higher than needed for maintenance of mtDNA copy number. However, almost 95 percent of the replication events terminate prematurely resulting in formation of the 7S DNA [21]. Specific conserved short sequences have been identified that are associated with this premature termination event and are referred to as TAS and ETAS (extended TAS) elements [11], [22]. It has been shown that this replication termination might regulate the mtDNA copy number [23], [24]. The levels of mtDNA within a cell change according to the oxidative needs and, coupled with transcription, defines the oxidative capacity of the cell. Eight variant nucleotide positions within the D-loop were located in known functional sites. However, analysis of mitochondrial D-loop sequences from 27 mammalian species revealed a length variation in the ETAS sequences [25]. Moreover, in the human mtDNA two regions, HV1 and HV2, have been shown to be hypervariable [26], [27]. HV1 in human mtDNA corresponds to positions 15284−15643 in the rat and hence, the variant nucleotide positions at these locations might not lead to major functional changes (Table 1). However, the D-loop variations located within the central block (CB) and conserved sequence block 2 (MT-CSB2) might affect mitochondrial biogenesis, since they are located outside the two hypervariable regions.

The results of the neutrality tests did not provide obvious evidence for selection in any of the non-protein-coding regions. Only Tajima's D test provided evidence for selection in *16S rRNA* and *tRNA-Cys*. The disagreement between the tests is likely caused by the different approaches employed to identify deviation from neutrality. The two Fu & Li's tests consider the genealogy of the sequences used to estimate the statistics, while the Tajima's test is genealogy independent. Considering the different sensitivities of the neutrality tests to the number of variable sites, these results must be interpreted with caution. Moreover, due to high mutation rate in mtDNA, especially in the D-loop, it is not possible to account for reverse-mutations, and hence we cannot completely rule out selection with the methods used. It should also be considered that the results presented here are based on analysis of 23 inbred strains and only four sequences from wild rats. In conclusion, we have identified a few sites in the RNA genes and the D-loop that might play a role in mitochondrial biogenesis and maintenance.

## Materials and Methods

### Sequences and Analysis

Twenty seven complete *Rattus norvegicus* mtDNA sequences available in public databases were used, 13 of which have been sequenced in our laboratory (Table S1). The wild rats included in the study were caught at different geographical locations – Wild/Swe (Malmö, Sweden), Wild/Mcwi (Milwaukie, USA), Wild/Cop (Copenhagen, Denmark), and Wild/Tku (Tokyo, Japan). Total genomic DNA extracted from rat tail was used to PCR amplify mtDNA with 32 overlapping primer pairs (Table S3). PCR products were cleaned with ExoSAP-IT (USB Corporation). Cycle sequencing was performed using BigDye (Applied Biosystems) followed by ethanol-EDTA precipitation and separation on ABI3730 DNA Sequencer (RSKC-Malmö core facility). The sequences were processed with Phred [28], [29] to assign quality values to each base call and assembled with the STADEN software [30].

### Comparative Sequence Analysis of the Non-Coding Mitochondrial DNA

Multiple sequence alignments were computed using ClustalX [31] and visually inspected. DnaSP v. 4.50.3 [32] was used to estimate the nucleotide statistics (segregating sites, haplotypes, nucleotide diversity). Since no crystal structure data are available for mammalian mitochondrial ribosomal RNAs, we referred to the predicted models for the mammalian mitoribosome [6], [33], [34]. Selected rat mitochondrial tRNA secondary structures were modeled on the predicted mammalian mitochondrial tRNA structures [8]. To assess the impact of variations in the RNA genes Mfold web server was used to compute the minimum free energy structures [35].

### Tests to Identify Selection

Tajima's D test [36], Fu & Li's D and F tests with outgroup [37] were performed using DnaSP v. 4.50.3 [32]. For the Fu & Li's tests, we used the mouse reference mtDNA sequence (NC_005089) as outgroup. All these tests assess whether the DNA sequence is evolving randomly (neutrally) or by a non-random process. Non-random processes stand for either directional selection or balancing selection. However, non-random events might also be due to changes in the population size. To assess the effect of population changes we estimated the F_S_ [38] and R2 [39] statistics using the DnaSP program. Fu's F_S_ test estimates population changes by considering the number of different haplotypes in the sample, while the R2 compares the difference between the number of singleton mutations and the average number of nucleotide differences. In lieu of coalescence based permutation tests, the selection tests were assessed for sensitivity to the number of segregating sites. Both the Fu & Li's tests were considerably less sensitive to the number of segregating sites modeled on the dataset. Since the wild rat sequences were quiet divergent from the inbred population we did not include these wild rat sequences in the neutrality tests.

## Supporting Information

Table S1 Rat strains and mtDNA sequences
(0.04 MB DOC)

Table S2 Variable positions in the rat non-protein-coding mtDNA
(0.06 MB XLS)

Table S3 Primer pairs used for PCR and DNA sequence analysis
(0.05 MB DOC)

## References

[1] Dyall SD, Brown MT, Johnson PJ (2004). Ancient invasions: from endosymbionts to organelles.. Science.

[2] Blier PU, Dufresne F, Burton RS (2001). Natural selection and the evolution of mtDNA-encoded peptides: evidence for intergenomic co-adaptation.. Trends Genet.

[3] Wu Z, Puigserver P, Andersson U, Zhang C, Adelmant G (1999). Mechanisms controlling mitochondrial biogenesis and respiration through the thermogenic coactivator PGC-1.. Cell.

[4] Scarpulla RC (2006). Nuclear control of respiratory gene expression in mammalian cells.. J Cell Biochem.

[5] Castillo-Davis CI, Hartl DL, Achaz G (2004). Cis-regulatory and protein evolution in orthologous and duplicate genes.. Genome Res.

[6] Mears JA, Sharma MR, Gutell RR, McCook AS, Richardson PE (2006). A structural model for the large subunit of the mammalian mitochondrial ribosome.. J Mol Biol.

[7] Springer MS, Douzery E (1996). Secondary structure and patterns of evolution among mammalian mitochondrial 12S rRNA molecules.. J Mol Evol.

[8] Helm M, Brule H, Friede D, Giege R, Putz D (2000). Search for characteristic structural features of mammalian mitochondrial tRNAs.. RNA.

[9] Cantatore P, Daddabbo L, Fracasso F, Gadaleta MN (1995). Identification by *in organello* footprinting of protein contact sites and of single-stranded DNA sequences in the regulatory region of rat mitochondrial DNA. Protein binding sites and single-stranded DNA regions in isolated rat liver mitochondria.. J Biol Chem.

[10] Roberti M, Musicco C, Polosa PL, Milella F, Gadaleta MN (1998). Multiple protein-binding sites in the TAS-region of human and rat mitochondrial DNA.. Biochem Biophys Res Commun.

[11] Sbisa E, Tanzariello F, Reyes A, Pesole G, Saccone C (1997). Mammalian mitochondrial D-loop region structural analysis: identification of new conserved sequences and their functional and evolutionary implications.. Gene.

[12] Larizza A, Pesole G, Reyes A, Sbisà E, Saccone C (2002). Lineage specificity of the evolutionary dynamics of the mtDNA D-loop region in rodents.. J Mol Evol.

[13] Patel VB, Cunningham CC, Hantgan RR (2001). Physiochemical properties of rat liver mitochondrial ribosomes.. J Biol Chem.

[14] Smits P, Smeitink JAM, van den Heuvel LP, Huynen MA, Ettema TJG (2007). Reconstructing the evolution of the mitochondrial ribosomal proteome.. Nucl Acids Res.

[15] Kraft A, Lutz C, Lingenhel A, Grobner P, Piendl W (1999). Control of ribosomal protein L1 synthesis in mesophilic and thermophilic archaea.. Genetics.

[16] Mayer C, Kohrer C, Grobner P, Piendl W (1998). MvaL1 autoregulates the synthesis of the three ribosomal proteins encoded on the MvaL1 operon of the archaeon *Methanococcus vannielii* by inhibiting its own translation before or at the formation of the first peptide bond.. Mol Microbiol.

[17] Branlant C, Krol A, Machatt A, Ebel J-P (1981). The secondary structure of the protein L1 binding region of ribosomal 23S RNA. Homologies with putative secondary structures of the L11 mRNA and of a region of mitochondrial 16S rRNA.. Nucleic Acids Res.

[18] Hixson JE, Wong TW, Clayton DA (1986). Both the conserved stem-loop and divergent 5′-flanking sequences are required for initiation at the human mitochondrial origin of light- strand DNA replication.. J Biol Chem.

[19] Tapper DP, Clayton DA (1981). Mechanism of replication of human mitochondrial DNA. Localization of the 5′ ends of nascent daughter strands.. J Biol Chem.

[20] Martens PA, Clayton DA (1979). Mechanism of mitochondrial DNA replication in mouse L-cells: localization and sequence of the light-strand origin of replication.. J Mol Biol.

[21] Bogenhagen D, Clayton DA (1978). Mechanism of mitochondrial DNA replication in mouse L-cells: kinetics of synthesis and turnover of the initiation sequence.. J Mol Biol.

[22] Doda JN, Wright CT, Clayton DA (1981). Elongation of displacement-loop strands in human and mouse mitochondrial DNA is arrested near specific template sequences.. Proc Natl Acad Sci U S A.

[23] Brown TA, Clayton DA (2002). Release of replication termination controls mitochondrial DNA copy number after depletion with 2′,3′-dideoxycytidine.. Nucleic Acids Res.

[24] Kai Y, Miyako K, Muta T, Umeda S, Irie T (1999). Mitochondrial DNA replication in human T lymphocytes is regulated primarily at the H-strand termination site.. Biochim Biophys Acta.

[25] Sbisa E, Tanzariello F, Reyes A, Pesole G, Saccone C (1997). Mammalian mitochondrial D-loop region structural analysis: identification of new conserved sequences and their functional and evolutionary implications.. Gene.

[26] Ruiz-Pesini E, Lott MT, Procaccio V, Poole JC, Brandon MC (2007). An enhanced mitomap with a global mtDNA mutational phylogeny.. Nucleic Acids Res.

[27] Stoneking M (2000). Hypervariable sites in the mtDNA control region are mutational hotspots.. Am J Hum Genet.

[28] Ewing B, Green P (1998). Base-calling of automated sequencer traces using Phred. II. Error probabilities.. Genome Res.

[29] Ewing B, Hillier L, Wendl MC, Green P (1998). Base-calling of automated sequencer traces using Phred. I. Accuracy assessment.. Genome Res.

[30] Staden R (1996). The staden sequence analysis package.. Mol Biotechnol.

[31] Larkin MA, Blackshields G, Brown NP, Chenna R, McGettigan PA (2007). Clustal W and Clustal X version 2.0.. Bioinformatics.

[32] Rozas J, Sanchez-DelBarrio JC, Messeguer X, Rozas R (2003). DnaSP, DNA polymorphism analyses by the coalescent and other methods.. Bioinformatics.

[33] Cavdar Koc E, Burkhart W, Blackburn K, Moseley A, Spremulli LL (2001). The small subunit of the mammalian mitochondrial ribosome. Identification of the full complement of ribosomal proteins present.. J Biol Chem.

[34] Burk A, Douzery EJP, Springer MS (2002). The secondary structure of mammalian mitochondrial 16S rRNA molecules: refinements based on a comparative phylogenetic approach.. J Mamm Evol.

[35] Zuker M (2003). Mfold web server for nucleic acid folding and hybridization prediction.. Nucl Acids Res.

[36] Tajima F (1989). Statistical method for testing the neutral mutation hypothesis by DNA polymorphism.. Genetics.

[37] Fu YX, Li WH (1993). Statistical tests of neutrality of mutations.. Genetics.

[38] Fu YX (1997). Statistical tests of neutrality of mutations against population growth, hitchhiking and background selection.. Genetics.

[39] Ramos-Onsins SE, Rozas J (2002). Statistical properties of new neutrality tests against population growth.. Mol Biol Evol.

[40] Gibbs RA, Weinstock GM, Metzker ML, Muzny DM, Sodergren EJ (2004). Genome sequence of the Brown Norway rat yields insights into mammalian evolution.. Nature.

[41] Pak JW, Vang F, Johnson C, McKenzie D, Aiken JM (2005). MtDNA point mutations are associated with deletion mutations in aged rat.. Exp Gerontol.

[42] Schlick NE, Jensen-Seaman MI, Orlebeke K, Kwitek AE, Jacob HJ (2006). Sequence analysis of the complete mitochondrial DNA in 10 commonly used inbred rat strains.. Am J Physiol Cell Physiol.

[43] Nilsson MA, Gullberg A, Spotorno AE, Arnason U, Janke A (2003). Radiation of extant marsupials after the K/T boundary: evidence from complete mitochondrial genomes.. J Mol Evol.

[44] Gadaleta G, Pepe G, De Candia G, Quagliariello C, Sbisa E (1989). The complete nucleotide sequence of the Rattus norvegicus mitochondrial genome: cryptic signals revealed by comparative analysis between vertebrates.. J Mol Evol.

