# Supplementary material for: Comparative Sequence Analysis of the Non-Protein-Coding Mitochondrial DNA of Inbred Rat Strains
Source: PLoS One. 2009 Dec 7;4(12):e8148. doi: 10.1371/journal.pone.0008148 (PMC2781161; doi:10.1371/journal.pone.0008148)
Supplement: Table S1 — Rat strains and mtDNA sequences (0.04 MB DOC) [file pone.0008148.s001.doc]

Table S1

Rat strains and mtDNA sequences.

| **Strain Name** | **Accession number** | **Source** |
| --- | --- | --- |
| **ACI/Eur** | DQ673908 | [42] |
| **B** | FJ919763 | This publication |
| **BBDP/Rhw** | FJ919760 | This publication |
| **BBDR/Rhw** | FJ919771 | This publication |
| **BN/SsNHsd/MCW** | NC_001665 | [40] |
| **F344 x BN F1** | AY769440 | [41] |
| **F344/DuCrl2Swe** | FJ919761 | This publication |
| **F344/NHsd** | DQ673909 | [42] |
| **FHH/Eur** | DQ673910 | [42] |
| **GH/OmrMcwi** | DQ673911 | [42] |
| **GK/Far** | DQ673912 | [42] |
| **GK/KyoSwe** | FJ919766 | This publication |
| **GK/Swe** | DQ673913 | [42] |
| **L4** | FJ919764 | This publication |
| **Lew/Ztm** | FJ919759 | This publication |
| **MHS/Gib** | FJ919770 | This publication |
| **PVG/OlaHsd** | FJ919762 | This publication |
| **SHR/mol** | FJ919768 | This publication |
| **SHRSP** | FJ919769 | This publication |
| **SS/JrHsd/Mcwi** | DQ673914 | [42] |
| **T2DN/Mcwi** | DQ673915 | [42] |
| **Wild/Cop** | AJ428514 | [43] |
| **Wild/Mcwi** | DQ673916 | [42] |
| **Wild/Swe** | FJ919765 | This publication |
| **Wild/Tku** | DQ673917 | [42] |
| **WK/Kyoto** | FJ919767 | This publication |
| **WKY/NCrl** | DQ673907 | [42] |
