# Supplementary material for: Comparative Sequence Analysis of the Non-Protein-Coding Mitochondrial DNA of Inbred Rat Strains
Source: PLoS One. 2009 Dec 7;4(12):e8148. doi: 10.1371/journal.pone.0008148 (PMC2781161; doi:10.1371/journal.pone.0008148)
Supplement: Table S3 — Primer pairs used for PCR and DNA sequence analysis (0.05 MB DOC) [file pone.0008148.s003.doc]

Table S3

Primer pairs used for PCR and DNA sequence analysis

| Nr. | Forward | Reverse |
| --- | --- | --- |
| 1 | AGGTTTGGTCCTGGCCTTAT | TGGAATTTTTGAGGTTTGTGG |
| 2 | ACCTAAGCCCAATAACGA | GGCCAGATGCTTTGTGAT |
| 3 | ATGAGAGGAGATAAGTCGTA | GAATTTTTGAGGTTTGTGGA |
| 4 | AGCCAAAAGAGGGACAGCTC | AGGGCTCCGAATAGGGAGTA |
| 5 | TTATTAGGGTGGCAGAGC | TGATGTTAGGGCGTTTAT |
| 6 | TCTAGCCACATCCAGCCTTT | GGGGGAGTCCTCCTAGTGAT |
| 7 | TTCAAAATTCTCCGTGCC | TTCGTGTTTGGGTCTGGT |
| 8 | ATTTCATCAGTCTTTGTT | TCCGATATCTTTGTGGTT |
| 9 | CACACTCTCACTCGCATGAA | GGCCTACTGTGAATATGTGATGTG |
| 10 | GGCTCAACCCCTGTCTTT | ACCTGCTGCTAATACTGG |
| 11 | CACGAATAAATAACATAAGC | ATAAGAAGATAAACCCTAAG |
| 12 | AATTCTTCCAGGGTTTGGAAT | ACGCTCTAAGCTTCGCAATG |
| 13 | GCCTTAGGGTTTATCTTC | GCGTCTATTGTGCTTGTG |
| 14 | TTAAAAACCGACGCAATC | GTGGGGGTAATGAAAGAG |
| 15 | CCGCCTAAACCAAGCTACAG | GAGCCGTAAATTCCGTCTGA |
| 16 | TCAGAACGCCTAATCAGCA | TGTGGTGGCCTTGGTATGT |
| 17 | CAAGCCTATGTATTCACC | GTTTGCTTTTTCGGAGTA |
| 18 | TTACATGAGCCCATCACAGC | TGGATTGAAATGAGGGCAAT |
| 19 | TTCATAGCAACGGGATTTC | CTAGGCAGAGGAGAGTGGA |
| 20 | AGCCTTTACATTATCTCTA | GTTTGGTTGCCTCATCGTG |
| 21 | TACACGATGAGGCAACCAAA | TCCTACCCCTTCTCATCCAA |
| 22 | GGCTTAACCTCATCACTCT | GTTCTTGCATACTTTCTTG |
| 23 | ACAGCGGCCCTCCAAGCAA | AATGTGGAGGAAAGCAAGG |
| 24 | ACCCCCACATTAACCGATTC | GATGTTGGGGTTATGTTGGA |
| 25 | TGCCCTACTACACTCAAGC | GGCTCAGGCGTTGGTGTTA |
| 26 | CCTAGGATTTGCCATTGC | CTGCTATGGCTACTGAGG |
| 27 | CCCAGCCACCACTATCATTC | TGATGGAGGCTAGTTGACCA |
| 28 | CATCAGTCACCCACATCT | GGTGTTGAGGGGGTTAGC |
| 29 | GATCAAATAACCCCACAG | CTTTGGGTGTTGATGGTG |
| 30 | CCTTCCTACCATTCCTGCAT | TGCTTGATGCCCTCTCCTTA |
| 31 | CACCATCAACACCCAAAGC | GGCCCTGAAGTAAGAACCA |
| 32 | AATTAAATAAAACAAAAAGC | TATAAAGTACCGCCAAGTCC |
